# Optimal Drop Height in Prepubertal Boys Is Revealed by the Performance in Squat Jump

**DOI:** 10.3390/sports11010001

**Published:** 2022-12-21

**Authors:** Eleni Bassa, Ilias Adamopoulos, Vassilios Panoutsakopoulos, Anthi Xenofondos, Athanasios Yannakos, Christos Galazoulas, Dimitrios A. Patikas

**Affiliations:** 1 Laboratory of Evaluation of Human Biological Performance, Department of Physical Education and Sport Science at Thessaloniki, Aristotle University of Thessaloniki, 54124 Thessaloniki, Greece; 2 Biomechanics Laboratory, School of Physical Education and Sports Sciences at Thessaloniki, Aristotle University of Thessaloniki, 54124 Thessaloniki, Greece; 3 Department of Education, Frederick University, Limassol 3080, Cyprus; 4 School of Physical Education and Sport Science at Serres, Aristotle University of Thessaloniki, 62110 Serres, Greece

**Keywords:** vertical jump, development, age differences, stretch-shortening cycle, performance, ground reaction forces, kinematics, kinetics, individuality, optimization

## Abstract

Drop jump (DJ) performance gain with increasing drop height is well documented in adults, but there is still no clear evidence of such gain in children. This study aimed to examine the differences in DJ performance gain in male adults and prepubescent boys by comparing drop heights tailored to each individual’s performance and expressed as a percentage of their squat jump (SJ) performance. Fifteen boys (9–11 y) and 15 men (19–27 y) executed DJs from drop heights that were set at 75%, 100%, 125%, and 150% of their best performance in SJ (DJ_75_, DJ_100_, DJ_125_, and DJ_150_, respectively). Vertical ground reaction force (vGRF), contact time and kinematics of the lower extremities were captured. The results showed that boys jumped significantly lower than adults in DJs, and both age groups presented jumping gain with increasing drop height, up to DJ_125_. Boys demonstrated longer total contact time, lower angular velocity and vGRF during the propulsive phase, as well as smaller knee flexion at touchdown and lower reactive strength index. vGRF in DJ_75_ and DJ_100_ was lower than in DJ_125_ and DJ_150_. The highest value for maximum knee flexion was also presented at DJ_150_. It is concluded that in prepubescent boys, the appropriate drop height for an effective DJ is linked to their performance in SJ and might be between 75% and 125% of their maximum SJ performance.

## 1. Introduction

Squat jump (SJ), countermovement jump (CMJ), and drop jump (DJ) are commonly used as tests to evaluate the power output capabilities of the lower limbs [1]. Comparing the outcome of these tests provides a better understanding of the contribution of the stretch-shortening cycle (SSC), which comprises elastic energy storage during the eccentric phase and later energy release during the subsequent concentric contraction [2,3]. Untrained adults present higher performance during CMJ compared with SJ (referred to as jumping gain) due to the prestretch that occurs to a greater extent during the eccentric phase of the CMJ [4]. When dropping from a height, as is the case in DJ, prestretch increases, and this may augment further the positive work of SSC, resulting in an even higher jumping gain [5,6].

The external load exerted on the body during a DJ is determined to a great extent by the drop height, and this defines the intensity of a DJ [7,8,9]. There are some controversial findings concerning the effect of drop height on performance. Some studies report no change in DJ performance when increasing drop height [10,11,12]. In contrast, others argue that in both trained and untrained adults, jumping performance increases up to an optimal drop height, and then a plateau appears [13,14], followed by a reduction in jumping performance when drop height increases further [2,8,15]. One possible reason for this disagreement could be the selection of non-individualized drop heights, if it is assumed that each participant has a different optimal drop height.

The effect of fixed (not individualized) drop heights on DJ performance has received much attention during the last decade in both children [16,17] and adults [18,19]. During the developmental period, jumping performance is affected by age. Younger children demonstrate more jumping gain than older children when gain is defined as the difference in performance between CMJ and SJ jumping height [20]. However, this decreased gain in older children is likely attributed to their increased SJ performance as they grow up [21]. Relevant information concerning DJ in children is limited. It has been previously reported that children present no gain in performance during DJ compared with CMJ [16,17,22]. The same finding was also observed in adult sportsmen [23]. Furthermore, the lower DJ performance of children compared with adults was attributed to their lower values in preactivation level, stretch reflex, agonist activation, and musculotendinous stiffness [24]. Additionally, children seem more resistant to physiological adaptations after plyometric training compared with adults. More specifically, plyometric training improves adults’ DJ performance with a more efficient SSC, as they increase their musculotendinous stiffness [25]. On the contrary, children whose musculotendinous unit is more compliant [26,27,28] increase their jumping performance after the implementation of plyometric training by flexing their knee joints more and, therefore, increasing the distance that the center of mass accelerates during the concentric phase of the drop jump [29]. This adaptation was attributed to the different loads the neuromuscular system can handle during development [16,17].

Even fewer studies are available concerning the effect of drop height on children’s jumping performance. Although the ability of children to use the SSC effectively has been confirmed in boys and girls during low impact jumps by demonstrating gain in CMJ performance compared with SJ [22], this was not the case for DJ from different drop heights. No difference in performance and DJ kinetics was found across dropping heights for either boys or girls [16,17]. Neither prepubertal [24,30] nor pre-, circa-, or post-pubertal boys and girls [31] presented any gain in the performance of DJs from various drop heights when compared with CMJ [16,17]. This indicates possibly a reduced capacity of children to use the SSC efficiently when stretch velocity is increased [16,17]. As mentioned above, DJ intensity increases as drop height increases. It has been argued that children presented no DJ gain when executing DJs from 20 and 40 cm compared with SJ [24]. This was attributed to the fact that children had likely exceeded the level of intensity (i.e., stretching velocity) that they could afford, whereas this was not the case for adults who were tested for the same drop heights [24].

Using common drop heights for individuals of different age groups may seem rational for extracting conclusions regarding matters of everyday life (e.g., the height of a step of a stair is the same for people of any age). However, the conclusion about the lack of DJ gain in children might be misleading, considering the inherent differences in body size (height, muscle mass) and, consequently, strength capacity [32]. Therefore, adjusting the drop height to a property related to each individual’s size or strength might reveal a gain in performance when increasing the drop height and may indirectly reveal the contribution of SSC even during DJ in children, as observed in adults.

Individualization is a key training principle, since adjusting the intensity to each individual’s requirements may lead to more optimal adaptations [33]. In male trained athletes, DJ performance from an individualized drop height, defined as a percentage of each person’s maximum vertical CMJ height, showed an optimal drop height either lower than 50% or between 50% and 125% of maximum CMJ height [10,11]. To our knowledge, there are no studies examining this issue in children. Therefore, the current study was designed to document the possible differences in DJ performance gain among drop heights equal to 75%, 100%, 125%, and 150% of performance in SJ in untrained boys and men. To explain these differences, the lower limb kinematics and vertical ground reaction force (vGRF) were recorded. It was hypothesized that individualized drop height will reveal jumping gain in DJ for both age groups. The information derived from this study will help coaches to better predict the optimal drop height for different age groups, which is crucial for both training and testing.

## 2. Materials and Methods

### 2.1. Participants

Thirty males (15 prepubescents, 15 adults) volunteered to participate in this study. Prepubescent boys were 9–11 years old (age: 10.50 ± 0.64 years, body mass: 40.79 ± 5.51 kg, and body height: 144.79 ± 5.81 cm), and men were 19–27 years old (age 24.07 ± 3.41 years, body mass: 80.13 ± 8.63 kg, and body height: 179.87 ± 5.54 cm). According to Tanner’s criteria, children were classified as prepubertal (Stage 1) by the same physician [34]. Both boys and men were untrained and did not participate systematically in any sports training program during the past two years. They were healthy and did not suffer from any chronic disease (cardiovascular disease, diabetes, hypertension, arthritis, obesity, etc.), they were free of any neurological symptom that could influence the lower extremity motor output, and they had no history of back or a lower limb injury. Before testing, all adult participants and the parents of the prepubescent boys read and signed a written informed consent statement. The study was planned in accordance with the guidelines of the Declaration of Helsinki, updated in Fortaleza (World Medical Association Declaration of Helsinki: Ethical principles for medical research involving human subjects) [35], met the ethical standards of Aristotle University of Thessaloniki, Greece, and was approved by an Institutional Reviewing Board (approval code: 1/24.10.2013).

### 2.2. Experimental Procedure

Initially, the anthropometric characteristics were measured (Table 1). Body mass was measured to the nearest 0.1 kg using a digital scale (BC-543, TANITA, Tokyo, Japan), and body height was measured to the nearest 0.1 m using a stadiometer (Bodymeter 206, Seca, Ningbo, China). Then, the participants followed a warm-up program, including 5 min treadmill walking–jogging of low to medium tempo, followed by ten repetitions of 6 dynamic stretching exercises (hip in, hip out, knee hugs, heel kicks, forward, and lateral leg swings). A familiarization session followed, performing submaximal SJ, CMJ, and DJ from 5–20 cm.

After the placement of the markers, the participants performed 3 maximal SJs by flexing slowly their knees at 90°, as measured with a manual goniometer, and maintaining this position for approximately 2 s before jumping as high as possible. The highest jump height was used for the calculation of the drop heights. The DJ test included DJs from 75%, 100%, 125%, and 150% of each participant’s maximum SJ height (DJ_75_, DJ_100_, DJ_125_, and DJ_150_, respectively). Individualized drop heights were attained with a precision of 1 cm by using combinations of custom-made wooden boxes (height of 5, 10, and 20 cm) and plates (height of 1 and 3 cm).

The DJ was initiated by stepping forward from the drop platform (without pushing or hopping) with the leg of preference [36]. Any landing that required extra steps or hops after touch-down was repeated. The participants started each jump standing akimbo and were barefooted. No arm swing was allowed at any phase of the DJ. Three trials were performed from each drop height. The interval was 1 min between each trial and 3 min between each drop height. The order of the drop heights was randomized and all measurements were assessed by one investigator.

### 2.3. Instrumentation

Kinematic data were captured using a six-camera 3D motion analysis system (VICON 612, Oxford Metrics Ltd., Oxfordshire, UK), with a sampling frequency set at 100 Hz. To obtain the kinematics, 16 reflective spherical markers (14 mm diameter) were placed at anatomical bony landmarks of the lower body according to the Helen Hayes model [37]. Vicon system’s high reliability, reproducibility, and validity was previously reported [38,39]. vGRFs were recorded with a ground-mounted 40 × 60 cm force plate (Bertec Type 4060, Bertec Corporation, Columbus, OH, USA) operating with a sampling rate of 1 kHz. Prior to data collection, the motion analysis system was calibrated according to the manufacturer’s recommendations and was used to synchronize all signals.

### 2.4. Data Analysis

Data were processed offline using Matlab R2021 scripts (The MathWorks Inc., Natick, MA, USA). Jump height was estimated from the vGRF–time curve, as described in a previous study [40]. Only the best trial in terms of jump height was further analyzed.

The performance gain was calculated as presented in Equation (1) [41]:

(1) $$\mathrm{Gain}\;\left(\%\right)=\left(\frac{{\mathrm{DJ}}_{\mathrm{height}}}{{\mathrm{SJ}}_{\mathrm{height}}}\;-\;1\right)\;\times \;100$$

Furthermore, the reactive strength index (RSI) of the lower limbs during the DJs was estimated according to Flanagan et al. [42], namely as the ratio of jump height to total contact time.

The knee joint angle was calculated at the following time-instances: at 100 ms and 50 ms before landing and at touchdown and at maximal knee flexion during the DJs. The duration of the braking phase (instance of contact with the ground until the instance of maximal knee flexion) and the propulsive phase (instance of maximal knee flexion until take-off) were evaluated, as well as the total contact time (instance of contact with the ground until take-off). The peak angular velocity of the knee joint during the braking and propulsion phase was evaluated. The peak vGRF was normalized to the body weight. Finally, the knee joint stiffness (k) was calculated as follows (Equation (2)) [43]:

(2) $$k=\frac{Μ}{\delta \theta }$$

where Μ is the knee joint moment when the knee angle was maximal, and *δ*θ is the joint angular displacement from touch-down to maximal knee flexion.

### 2.5. Statistical Analysis

Mean and *SD* was assessed for all dependent variables. Linear mixed-effects model analyses were conducted to determine the main effects of age group (factor AGE; 2 levels: boys and men) and drop height (factor HEIGHT; 4 levels: DJ_75_, DJ_100_, DJ_125_, and DJ_150_) as well as their interaction. AGE and HEIGHT were set as fixed factors and participants as a random factor. To explore significant differences between sub-groups, pairwise Bonferroni-adjusted comparisons were performed. Effect sizes were estimated by calculating partial eta squared (*η*^2^*_p_*), classified as small (0.01–0.05), medium (0.06–0.13), and large (≥0.14). All statistical analyses were performed using the R v.4.2.1 software (R Foundation for Statistical Computing, Vienna, Austria), and the significance level was set at *α* = 0.05.

## 3. Results

### 3.1. Squat Jump Performance

The height of the SJ was 30.00 ± 1.89 cm and 15.85 ± 2.37 cm for men and boys, respectively. Squat jump performance provided the basis for the definition of the dropping heights for DJ_75_, DJ_100_, DJ_125_, and DJ_150_.

### 3.2. Drop Jump Performance and Gain

**Figure 1:**
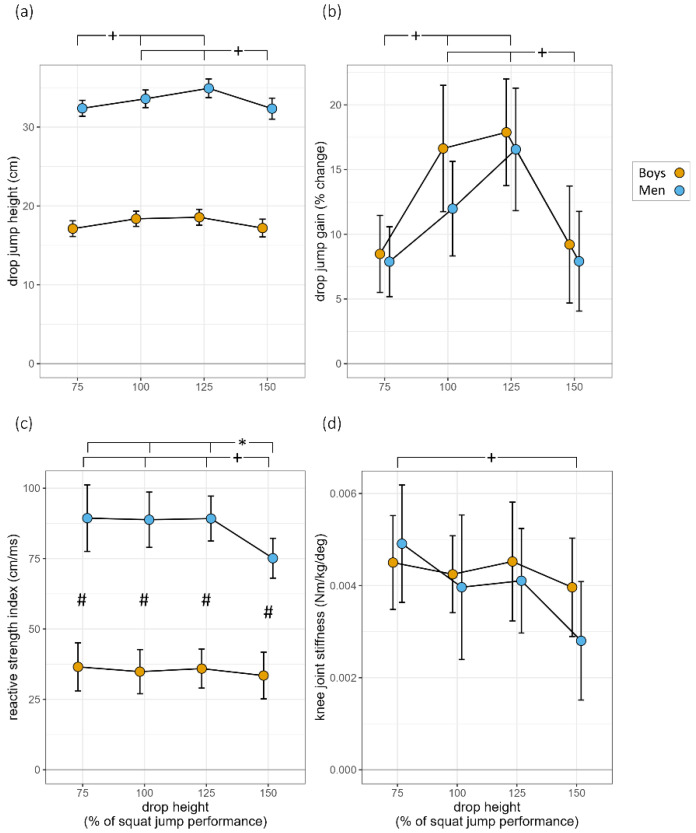
Results for the examined parameters: (**a**) drop jump height; (**b**) drop jump gain; (**c**) reactive strength index; and (**d**) knee joint stiffness. *NOTE*: #: significant (*p* < 0.05) AGE main effect; +: significant (*p* < 0.05) HEIGHT main effect; and *: significant (*p* < 0.05) AGE × HEIGHT interaction.

ANOVA revealed an effect of drop height on DJ performance (*F*_(3,84)_ = 19.2, *p* < 0.001, *η*^2^*_p_* = 0.41; Figure 1a). DJ_100_ and DJ_125_ jump performance was significantly higher than DJ_75_ (*p* < 0.05), while DJ_150_ was significantly lower than DJ_100_ and DJ_125_ (*p* < 0.05). Drop Jump height was significantly higher in men than in boys (*F*_(1,28)_ = 387.6, *p* < 0.001, *η*^2^*_p_* = 0.97). No significant interaction was detected (*p* > 0.05).

For the gain in DJ performance compared with SJ, a significant effect of drop height was found (*F*_(3,84)_ = 19.6, *p* < 0.001, *η*^2^*_p_* = 0.41) with increasing gain values up to DJ_125_ (Figure 1b). Post-hoc tests revealed significantly less gain at DJ_75_ compared with DJ_100_ and DJ_125_ (*p* < 0.001), while DJ_150_ was significantly lower than DJ_100_ and DJ_125_ (*p* < 0.001). No significant difference between groups (*p* > 0.05) nor interaction was revealed (*p* > 0.05).

### 3.3. Vertical Ground Reaction Force

During the braking phase, a significant effect of drop height on vGRF (*F*_(3,98)_ = 13.5, *p* < 0.001, *η*^2^*_p_* = 0.29) was found (Table 2). Vertical ground reaction force in DJ_75_ and DJ_100_ differed significantly from DJ_150_ (*p* < 0.001). Boys’ vGRF values, although lower, were not statistically different (*p* > 0.05) from those of men. No interaction for height × age was found (*p* > 0.05). During the propulsion phase, a significant effect of drop height was found in vGRF (*F*_(3,84)_ = 2.8, *p* < 0.05, *η*^2^*_p_* = 0.09), and boys generated lower vGRF than men (*F*_(1,28)_ = 13.4, *p* < 0.005, *η*^2^*_p_* = 0.49). No interaction of height × age was found (*p* > 0.05).

### 3.4. Contact Time

During the braking phase, a significant effect of drop height on contact time was revealed (*F*_(3,84)_ = 2.9, *p* < 0.05, *η*^2^*_p_* = 0.09). Boys presented statistically significant longer contact time than adults (*F*_(1,14)_ = 19.3, *p* < 0.001, *η*^2^*_p_* = 0.58). No significant interaction was detected (*p* > 0.05). During the propulsion phase, a significant effect of drop height was found in contact time (*F*_(3,84)_ = 12.9, *p* < 0.001, *η*^2^*_p_* = 0.31), as contact time increased significantly at DJ_150_ (*p* < 0.001). No difference between groups nor interaction of drop height × group was found (*p* > 0.05). The total contact time changed at different drop heights (*F*_(3,84)_ = 3.7, *p* < 0.05, *η*^2^*_p_* = 0.12), and boys presented longer total contact time than men (*F*_(1,28)_ = 8.4, *p* < 0.05, *η*^2^*_p_* = 0.37). No interaction of drop height × age was found for total time (*p* > 0.05).

### 3.5. Knee Angular Velocity

Table 3 presents the results for the knee angular kinematics. During the breaking phase, no significant effect of drop height on angular velocity was found (*p* > 0.05). Neither group difference nor significant interaction was found (*p* > 0.05). During the propulsion phase, a significant effect of drop height was found in knee angular velocity (*F*_(3,84)_ = 3.5, *p* < 0.05, *η*^2^*_p_* = 0.11), as both groups decreased their angular velocity at DJ_125_ compared with their angular velocity at DJ_100_ (*p* < 0.05). Boys generated lower angular velocity (*F*_(1,14)_ = 7.9, *p* < 0.05, *η*^2^*_p_* = 0.36) than men at DJ_100_, DJ_125_, and DJ_150_ (*p* < 0.05). No interaction was found (*p* > 0.05).

### 3.6. Knee Angle

No drop height effect was found for knee flexion at 100 ms and 50 ms before touchdown (*F*_(3,84)_ = 0.8, *p* = 0.507, *η*^2^*_p_* = 0.03 and *F*_(3,84)_ = 1.3, *p* = 0.288, *η*^2^*_p_* = 0.04, respectively). Boys’ knee flexion values at both time instances were not different from those of men (*F*_(1,14)_ = 0.5, *p* = 0.491, *η*^2^*_p_* = 0.03 and *F*_(1,14)_ = 1.1, *p* = 0.319, *η*^2^*_p_* = 0.07, respectively). No drop height × age interaction was found (*F*_(3,84)_ = 2.1, *p* = 0.111, *η*^2^*_p_* = 0.07 and *F*_(3,84)_ = 0.9, *p* = 0.442, *η*^2^*_p_* = 0.03 for 100 ms and 50 ms prior the touchdown, respectively).

Regarding the knee flexion angle at touchdown (Table 2), no effect of drop height was found (*p* > 0.05). A significant interaction of drop height × age was found (*F*_(3,84)_ = 5.4, *p* < 0.005, *η*^2^*_p_* = 0.16), as boys did not alter their knee flexion at touchdown in different drop heights, whilst men altered their knee flexion when dropping from DJ_150_. A significant age effect was revealed, as boys presented lower flexion at touchdown than adults (*F*_(1,14)_ = 17.8, *p* < 0.001, *η*^2^*_p_* = 0.56) in drop heights DJ_75_, DJ_100_, and DJ_125_ (*p* < 0.001) but not in drop height DJ_150_ (*p* > 0.05).

### 3.7. Reactive Strength Index

A significant effect of drop height on RSI was found (*F*_(3,84)_ = 6.7, *p* < 0.001, *η*^2^*_p_* = 0.19; Figure 1c). Boys presented lower scores than men (*F*_(1,14)_ = 60.5, *p* < 0.001, *η*^2^*_p_* = 0.81). A significant interaction of drop height × age was also found (*F*_(3,84)_ = 3.4, *p* < 0.05, *η*^2^*_p_* = 0.11), as RSI scores at DJ_150_ were significantly lower than DJ_75_, DJ_100_, and DJ_125_ (*p* < 0.001) in adults but remained unchanged in boys (*p* > 0.05).

### 3.8. Knee Joint Stiffness

A significant effect of drop height on knee joint stiffness was found (*F*_(3,84)_ = 2.8, *p* = 0.048, *η*^2^*_p_* = 0.09; Figure 1d). However, neither a significant difference between groups nor an interaction was revealed (*p* > 0.05).

## 4. Discussion

In the present study, we showed that prepubescent boys jumped lower than men from all individualized drop heights, but both age groups presented DJ gain with increasing drop height, up to DJ_125_. Vertical ground reaction force in DJ_75_ and DJ_100_ was lower than in DJ_125_ and DJ_150_. In addition, knee flexion at the deepest point increased significantly in DJ_150_. Compared with men, boys demonstrated lower RSI, with longer contact time (total and during the braking phase), lower angular velocity and vGRF during the propulsive phase, as well as less knee flexion at touchdown.

In previous studies concerning children, no effect of drop height on jumping performance has been reported for prepubescent children [22,24,30] or boys or girls of all maturity levels [31], leading to the conclusion that children are unable to manage the load when performing DJs from fixed drop heights. This assumption was further supported by Gillen et al. [16], who reported that there is a growth-related inability of children to use the energy that is stored during the eccentric phase when the external load is increased during DJs. To the best of our knowledge, this is the first study that tests the effect of individualized drop height on DJ performance in prepubescent boys, presenting jumping gain during DJs, when increasing drop height from 75% to 125% of SJ height.

The lower jumping performance of boys compared with adults is in accordance with previous studies in both prepubescent [22,24] and adolescent boys [44]. It was reported that prepubescent boys achieve lower jump heights because of their immature DJ technique, characterized by a prolonged contact time [22,24] and lack of DJ gain. However, these differences could be attributed to the application of common drop heights for boys and adults, resulting in a disproportional loading for these age groups. In the present study, DJ gain was evident in both age groups using individualized drop heights (percent of SJ performance). However, some of the biomechanical age-related differences are still evident.

In adults, we observed an increase in jumping performance when increasing drop height from 75% to 125% of SJ performance. This is in contrast to studies showing no gain in DJ performance when using drop heights from 50% to 150% of maximum CMJ height [10,11]. This discrepancy may be due to methodological differences regarding the reference in the calculation of drop height (i.e., SJ vs. CMJ). CMJ height is higher than SJ due to the energy stored and released during SSC [45]. Therefore, the lack of DJ gain could be attributed to the already high values of CMJ. On the other hand, SJ is initiated from a static position (knees flexed at 90°, with no countermovement) and requires the development of pretension by muscle coactivation in order to uptake muscle slack [46,47]. Therefore, SJ could be considered a better and more sensitive indicator of neuromuscular capacity, with a minor contribution of the passive components that are involved in SSC.

Vertical ground reaction force is an important biomechanical feature during DJ [48] that determines the loading over the joint, which might be crucial for both performance enhancement and injury prevention. We observed that vGRF increased with drop height in both groups. This is in line with a study in adult volleyball players using individualized drop heights [11]. However, this contrasts with another study with prepubescent boys using fixed drop heights (20 and 40 cm) which documented no vGRF increase with increasing drop height [22]. Vertical ground reaction force may provide a surrogate evaluation for the strain experienced by the muscles and the bones during jumping activities [49]. Taking into consideration that in both groups vGRF is increased during DJ_125_ and DJ_150_ but jump performance increased up to DJ_125_, it could be suggested to use 125% of SJ drop height as a safety limit for drop height in children and adults, provided the appropriate DJ technique has been adopted.

The RSI has been previously considered a crucial marker for identifying the optimal drop height, and it has been used in plyometric training as an index to control the intensity and as feedback to the participants [33]. Furthermore, it has been suggested that optimizing drop heights on the basis of the highest RSI scores may maximize the effects of plyometric training in young soccer players [50]. By definition, RSI increases when contact time is reduced and/or when jump height is increased, whilst the disproportional increase in contact time to jump height results in a decrement in RSI. Therefore, it is important to define which of its two determinants has changed [51], especially when comparing groups. In this study, RSI scores decreased significantly for men during DJ_150_. The current decreased RSI in DJ_150_ may be explained by the significant decrease of DJ height and the decreasing trend for total contact time during DJ_150_. On the other hand, in adult volleyball players, no significant effect of relative drop height on RSI was reported, although a decreasing trend for RSI was noted above the drop height of 100% CMJ [10]. This could be attributed to the different training level of the participants of these studies since trained subjects may have the capacity to achieve shorter contact time and greater DJ height [52].

RSI is also highly correlated with musculotendinous stiffness [53]. In the present study, drop height had a significant effect on stiffness, presenting a reduction during DJ_150_. As the dropping height increases, vertical downward velocity increases and, thus, the neuromuscular system is required to compensate for the additional load during the absorption phase by altering the power output and stiffness regulation [54,55]. A key factor for the absorption of the eccentric loading is the capability of the knee extensor muscles to apply eccentric strength [56]. Thus, the present findings concerning the knee joint angle and angular velocity can be attributed to this factor. Lower stiffness values when performing DJs from higher drop heights is likely a protective mechanism, developed to absorb energy and ensure a safe landing, via reflex pathways involving the Golgi tendon organs [57] and neural inhibition that prohibits an injury on the muscle-tendon unit [58]. However, stiffness remained unchanged when increasing drop height (30, 45, and 60 cm) in adult basketball players [53]. This suggests that training might have moderated the above-mentioned protective mechanism, since trained subjects are more accustomed to performing jumps of high impact. In children, lower stiffness has been previously reported during DJ [59]. This was also supported by children’s lower pre-activation prior to landing and lower muscle activation during braking phase [24]. However, no significant age difference in knee angle 100 ms and 50 ms prior to landing (which are indirect indicators of preactivation) was revealed in this study. Therefore, children may handle more efficiently the eccentric loading imposed by the drop jump task when performed from individualized drop heights [31]. The fact that in the present study no age difference was detected supports the notion that individualized drop heights regulate the load around the knee joint in a similar manner for children and adults.

There are some limitations in the study. Although all participants were not athletes, and children followed only their typical physical education lesson, the level of physical activity that may influence performance has not been documented with a specialized questionnaire. Therefore, we cannot rule out possible variations either in jump performance or in biomechanical parameters among subjects regarding this issue. Moreover, as the participants were untrained adult and prepubescent males with no previous training experience in DJ exercises, our findings may not apply to females, athletes, or children of other maturation statuses, as they may behave differently during DJs of different—even individualized—drop heights, as previous research indicates [2]. Furthermore, since this is a cross-sectional study, a longitudinal study design using individualized drop heights may determine their suitability and applicability during training. This is also dictated by a recent narrative review that underlines the need for individualized testing and evaluation of the athletes when applying plyometric training protocols [60]. Furthermore, although the electromyographic evaluation was beyond the aim of this study, differences between children and adults may be explained by an inability of children to activate efficiently agonist and antagonist muscles in order to achieve better performance when executing tasks involving the SSC [24]. For understanding the underlying mechanisms, future research examining the effect of a prolonged drop jump training protocol using individualized drop heights along with electromyographic evaluation, in both trained and untrained children of both sexes, is suggested.

## 5. Conclusions—Practical Application

As far as we know, this is the first time that DJ performance is evaluated in prepubescent boys using individualized drop heights. In our view, the results of this study support our hypothesis that individual approach in jumping tests may reveal each participant’s ability to optimally use stretch-shortening cycle, regardless the age. Additionally, this study provides new evidence of DJ performance gain with increasing height in male adults and prepubescent boys, provided that drop heights are tailored to each individual’s SJ performance. These findings offer insights with regard to more optimal and individualized drop height settings in prepubescent boys and have the potential to inform coaches and practitioners when designing plyometric programs for training, injury prevention, or rehabilitation. According to our findings, individualized drop heights based on SJ performance are suggested to be used for testing and possibly during plyometric training of prepubescent boys, as they seem to be safer than fixed drop heights. Furthermore, drop height loadings within the range from 75 to 125% of SJ performance might be more effective during plyometric training, although more research is required to elucidate this issue regarding the optimal loading and its effectiveness in children of different maturation status, training level, or sex. However, biomechanical differences between the age groups should also be considered to tackle the age-related differences in DJ performance.

## Figures and Tables

**Table 1:** Age and anthropometric characteristics of the participants.

Parameter	Adults (*n* = 15)	Boys (*n* = 15)
Age (y)	24.07 ± 3.41	10.50 ± 0.64
Height (cm)	179.87 ± 5.54	144.79 ± 5.81
Body mass (kg)	80.13 ± 8.63	40.79 ± 5.51

**Table 2:** Mean (SD) values of the peak vertical ground reaction forces (vGRF) and the temporal parameters for the two groups during the drop heights that were set at 75%, 100%, 125%, and 150% of their best performance in SJ (DJ_75_, DJ_100_, DJ_125_, and DJ_150,_ respectively).

	DJ_75_		DJ_100_		DJ_125_		DJ_150_		
	Boys	Men	Boys	Men	Boys	Men	Boys	Men	
**Peak vGRF (times body weight)**									
Braking phase	2.73 (1.02)	1.81 (1.32)	2.66 (1.09)	2.39 (1.47)	3.48 (1.74)	2.58 (1.07)	3.97 (1.79)	3.11 (1.50)	Age: *p* > 0.05 Height: *p* < 0.001 Height × Age: *p* > 0.05
Propulsive phase	1.23 (0.23)	1.51 (0.26)	1.20 (0.20)	1.48 (0.23)	1.14 (0.21)	1.44 (0.17)	1.12 (0.23)	1.42 (0.19)	Age: *p* < 0.005 Height: *p* < 0.05 Height × Age: *p* > 0.05
**Contact time (ms)**									
Braking phase	297 (72)	246 (73)	336 (55)	243 (44)	340 (43)	250 (42)	345 (44)	253 (36)	Age: *p* < 0.001 Height: *p* < 0.05 Height × Age: *p* > 0.05
Propulsive phase	185 (38)	170 (55)	201 (52)	172 (60)	192 (57)	175 (42)	265 (95)	223 (48)	Age: *p* > 0.05 Height: *p* < 0.001 Height × Age: *p* > 0.05
Total	483 (98)	406 (106)	537 (89)	415 (87)	525 (71)	425 (75)	536 (106)	455 (58)	Age: *p* < 0.05 Height: *p* < 0.05 Height × Age: *p* > 0.05

**Table 3:** Mean (*SD*) values of the knee angular kinematics for the two groups during the drop heights that were set at 75%, 100%, 125%, and 150% of their best performance in SJ (DJ_75_, DJ_100_, DJ_125_, and DJ_150,_ respectively).

	DJ_75_		DJ_100_		DJ_125_		DJ_150_		
	Boys	Men	Boys	Men	Boys	Men	Boys	Men	
**Knee angular velocity (deg·s^−1^)**									
Braking phase	507 (164)	554 (163)	456 (211)	573 (149)	505 (169)	625 (146)	487 (229)	680 (271)	Age: *p* > 0.05 Height: *p* > 0.05 Height × Age: *p* > 0.05
Propulsive phase	621 (142)	776 (190)	523 (191)	739 (168)	613 (206)	800 (159)	552 (185)	815 (168)	Age: *p* < 0.05 Height: *p* > 0.05 Height × Age: *p* > 0.05
**Knee angle flexion (deg)**									
100 ms before touchdown	15.3 (13.1)	17.2 (10.6)	13.4 (12.1)	21.8 (13.5)	15.3 (12.2)	18.9 (11.4)	14.8 (12.0)	15.8 (13.6)	Age: *p* > 0.05 Height: *p* > 0.05 Height × Age: *p* > 0.05
50 ms before touchdown	26.5 (16.4)	18.9 (12.1)	24.4 (14.2)	22.2 (14.9)	24 (12.5)	17.7 (10.4)	23.8 (11.9)	16.6 (13.6)	Age: *p* > 0.05 Height: *p* > 0.05 Height × Age: *p* > 0.05
At touchdown	18.4 (9.6)	33.2 (5.7)	19.4 (8.6)	30.3 (5.1)	19.7 (5.9)	30.9 (6.5)	21.4 (6.5)	24.1 (9.7)	Age: *p* < 0.001 Height: *p* > 0.05 Height × Age: *p* > 0.05
Maximum knee flexion	69.3 (12.1)	77.6 (13.7)	79.7 (15.0)	78.4 (11.0)	74.7 (12.4)	77.6 (9.5)	82.4 (14.2)	87.5 (10.7)	Age: *p* > 0.05 Height: *p* < 0.001 Height × Age: *p* > 0.05

## Data Availability

The data presented in this study are available on reasonable request from the corresponding author.
